# 30 years of dengue fatal cases in Brazil: a laboratorial-based investigation of 1047 cases

**DOI:** 10.1186/s12879-018-3255-x

**Published:** 2018-07-27

**Authors:** Priscila Conrado Guerra Nunes, Ana Maria Bispo de Filippis, Monique Queiroz da Rocha Lima, Nieli Rodrigues da Costa Faria, Fernanda de Bruycker-Nogueira, Jaqueline Bastos Santos, Manoela Heringer, Thaís Chouin-Carneiro, Dinair Couto-Lima, Bianca de Santis Gonçalves, Simone Alves Sampaio, Eliane Saraiva Machado de Araújo, Juan Camilo Sánchez-Arcila, Flávia Barreto dos Santos, Rita Maria Ribeiro Nogueira

**Affiliations:** 1 Viral Immunology Laboratory (LIV), Oswaldo Cruz Institute, IOC, FIOCRUZ, Avenida Brasil, 4365. Manguinhos, Rio de Janeiro, Brazil; 2 Flavivirus Laboratory (LABFLA), Oswaldo Cruz Institute- FIOCRUZ, Avenida Brasil, 4365. Manguinhos, Rio de Janeiro, Brazil; 3 Hematozoa Transmittors Mosquitoes Laboratory, Oswaldo Cruz Institute- FIOCRUZ, Avenida Brasil, 4365. Manguinhos, Rio de Janeiro, Brazil

**Keywords:** Dengue, Fatal cases, Epidemiology, Laboratorial diagnosis, Brazil

## Abstract

**Background:**

Dengue viruses (DENV) have emerged and reemerged in Brazil in the past 30 years causing explosive epidemics. The disease may range from clinically asymptomatic infections to severe and fatal outcomes. We aimed to describe the epidemiological, clinical and laboratorial aspects of the dengue fatal cases received by a Regional Reference Laboratory, Brazil in 30 years.

**Methods:**

A total of 1047 suspected fatal dengue cases were received from 1986 to 2015 and analyzed in the Laboratory of Flavivirus, FIOCRUZ. Suspected cases were submitted to viral detection, serological and molecular methods for cases confirmation. Influence of gender, age, serotype and type of infection (primary/secondary) on death outcome, as well the interactions between serotype and age or infection and age and type of infection were also studied.

**Results:**

A total of 359 cases (34.2%) were confirmed and DENV-1 (11.1%), DENV-2 (43.9%), DENV-3 (32.8%) and DENV-4 (13.7%) were detected. Overall, fatal cases occurred more often in primary infections (59.3%, *p* = 0.001). However, in 2008, fatal cases were mainly associated to secondary infections (*p* = 0.003). In 2008 and 2011, deaths were more frequent on children and those infected by DENV-2 presented a higher risk for fatal outcome. Moreover, children with secondary infections had a 4-fold higher risk for death.

**Conclusions:**

Dengue is a multifactorial disease and, factors such as viral strain/serotype, occurrence of secondary infections and co-morbidities may lead to a severe outcome. However, the high dengue incidence and transmission during epidemics, such as those observed in Brazil may overwhelm and collapse the public health services, potentially impacting on increased disease severity and mortality.

## Background

Dengue fever is caused by any of the four distinct serotypes (DENV-1 to 4), belonging to the *Flavivirus* family. It is the most important arboviral diseases affecting humans worldwide and its global prevalence has grown dramatically in recent decades. About 100 million people are infected and 500,000 people develop severe dengue leading to about 70,000 deaths annually [1]. It poses a significant public health and economic burden in tropical and subtropical endemic regions [2, 3]. Over the last decades, both the incidence and severity of dengue in Central, the Caribbean and South America have increased significantly [4]. A recent estimate reported that the number of apparent dengue cases more than doubled every decade between 1990 to 2013, from 8.3 million in 1990 to 58.4 million in 2013 and, with an average of 9000 dengue fatal cases occurring per year [5].

Although most DENV infections are asymptomatic, the disease can also present a broad spectrum of clinical signs and symptoms, ranging from an acute undifferentiated febrile illness to severe and fatal outcomes. Fatal cases may occur in over 10% of cases and 90% of deaths occur in children under 15 years old [6]. However, in recent decades, dengue and severe dengue have become more frequent among adults [7]. If dengue is not treated properly, a small proportion of patients may develop life-threatening complications [8], however, with early recognition of the disease severity and intensive care, fatal outcomes can decrease from ∼10% to less than 1% among severe cases [9, 10].

Factors such as the occurrence of secondary infections with a heterologous serotype increase the risk of developing a more severe disease, however the infecting and genetic variation of DENV strain, presence of co-morbidities, ethnicity, age and the patient’s immune conditions, such as profound thrombocytopenia, may also contribute to a more severe case [7, 11–14]. The early diagnosis and immediate treatment are essential to reduce the mortality caused by DENV [15], however, about 70% of the infected patients may choose not to seek treatment or treat themselves [2].

In Brazil, since dengue introduction in early 80’s, more than ten million cases have been reported during successive epidemics, more critically occurred on 2002, 2008, 2010, 2013, 2014 and 2015, and when 150, 561, 656, 674, 475 and 986 fatal cases were confirmed, respectively [16]. Despite the increased mortality, not all cases progressing to a fatal outcome are diagnosed by the health services [17].

The spread of dengue in Brazil resulted in the establishment of a National Network for Dengue Diagnosis in the year of 1989 [18] which aimed to contribute for the disease surveillance in the country, an important tool to predict epidemics [19]. This Network consists of Regional Reference Laboratories responsible for all Brazilian regions [20] and includes the Laboratory of Flavivirus (LABFLA) IOC/FIOCRUZ, established since 1986 and which maintains a surveillance program in the State of Rio de Janeiro.

A review on dengue diagnosis and epidemiology by the Regional Reference Laboratory in 25 years has been published previously [19], however, despite the availability and richness of the fatal cases received in the last 30 years, no review nor detailed report were carried out. Here, we aimed to describe the epidemiological and laboratorial aspects of the dengue fatal cases received between 1986 and 2015.

## Methods

### Suspected dengue cases

Suspected dengue fatal cases (*n* = 1047) were received between March 1986 and December 2015 during an active surveillance program performed by the Laboratory of Flavivirus, IOC/FIOCRUZ, Regional Reference Laboratory for the Brazilian Ministry of Health, located in RJ. As a Regional Reference Laboratory, suspected cases are received as convenience sampling for diagnosis and the cases investigation has been approved by resolution number CSN196/96 from the Oswaldo Cruz Foundation Ethical Committee in Research (CEP 274/05). Suspected cases samples were received accompained by investigation records and questionnaires containing the patient’s demographic (age, gender, date of birth, address) and clinical (onset of disease and sign and symptoms) information.

Acute serum samples (up to the 7th day after the onset of the symptoms) were stored at − 70 °C and submitted for virus isolation, molecular methods reverse transcriptase polymerase chain reaction (RT-PCR), Real-time Reverse Transcriptase PCR (TaqMan) assay (qRT-PCR) and NS1 antigen capture ELISA. Convalescent samples (> 7 days of symptoms) were tested by the hemagglutination inhibition (HI) assay and by the anti-DENV IgM and IgG capture ELISA tests. From the 1047 fatal cases, 614 were collected in the acute phase (up to the 7th day of symptoms) and 233 were convalescent cases (> 7 days of symptoms). A paired sampling was available in 43 cases. In 290 cases, the information on the days of illness was not available. Despite this, all dengue suspected cases received in the Regional Reference Laboratory are tested by all methods, when sample volume is available.

### Virus isolation

Virus isolation was performed by inoculation into C6/36 *Aedes albopictus* cell line [21] and isolates were identified by indirect fluorescent antibody test (IFAT) using serotype-specific monoclonal antibodies [22]. The C6/36 *Aedes albopictus* cell line was kindly provided in many opportunitites during the study by Dr. Pedro Vasconcelos from the Evandro Chagas Institute, the National Reference Laboratory for Arboviruses for the Brazilian Ministry of Health.

### Immunoglobulin M (IgM) antibody capture ELISA (MAC-ELISA)

The in-house MAC-ELISA was carried out for dengue cases confirmation as described in Nogueira et al. [23]. Alternatively, the Panbio dengue IgM Capture ELISA (Panbio Diagnostics, Queensland, Australia) was used for the qualitative detection of anti-DENV IgM antibodies in serum for fatal case confirmation.

### Haemaglutination inhibition (HI) test

HI test was performed to characterize dengue infections as primary or secondary, as described in Clarke and Casals [24].

### Immunoglobulin G (IgG) antibody detection ELISA (IgG-ELISA)

The IgG-ELISA has been previously described by Miagostovich et al. [25] and was performed for to characterize infections as primary or secondary infections in replacement to the HI test for dengue cases previously confirmed by virus isolation, RT-PCR and/or MAC-ELISA.

### Dengue NS1 ag detection

The Platelia™ Dengue NS1 Ag-ELISA kit (Biorad Laboratories, Marnes-La-Coquette, France) was peeformed according to the manufacturer’s instructions. Additionally, we used the Dengue NS1 Ag STRIP (Bio-Rad Laboratories, Marnes-La-Coquette, França), an immunochromatographic test (ICT), according the manufacturer’s instructions.

### Immunohistochemistry

The immunohistochemistry assay was performed as described elsewhere [26].

### Viral RNA extraction

The viral RNA was extracted from samples using the QIAamp Viral RNA Mini kit (Qiagen) following the manufacturer’s instructions and stored at -70C.

### Dengue reverse transcriptase-nested polymerase chain reaction (RT-nested-PCR)

RT-PCR for detecting and typing DENV was performed as described previously by Lanciotti et al. [27].

### Real-time reverse transcriptase PCR (TaqMan) assay –qRT-PCR

The one-step real-time RT-PCR assay was performed as described previously by Johnson et al. [28] in the ABI Prism® 7500 Sequence Detection System (Applied Biosystems, Foster City, CA, USA).

### Matrix layout analysis of the laboratorial diagnostic methods

The matrix layout analysis representing all combination of techniques performed for dengue fatal cases investigation was done using UpsetR according to Lex et al. [29].

### Statistical analysis

The derived data was tabulated in appropriate worksheets using the SPSS 21st version Program and evaluated by t-Test and Anova Test.

We used logistic generalized linear models (GLM) with logit link function to study the influence of gender, age, serotype and type of infection (primary/secondary) on death outcome (death/alive outcome was coded as binary variable).

For this analysis, 4344 confirmed dengue cases that did not evolve to death were included. The inclusion criterion of those cases were non-fatal dengue suspected cases received between 1986 and 2015, confirmed by specific laboratorial diagnosis and with demographical information.

Interactions between serotype and age or infection and age and type of infection were also studied. Odd ratios (OR) were estimated from regression slope coefficients (β) calculating the OR = *e*^β^. Similarly, the 95% confidence interval (95%CI) for each OR was obtained through exponentiation of 95%CI estimated on the GLM. Due to the small number of individuals considered to evaluate the interaction of DENV-4 and the type of infection, the OR was not calculated. This analysis was performed using R statistical environment [30].

## Results

**Fig. 1:**
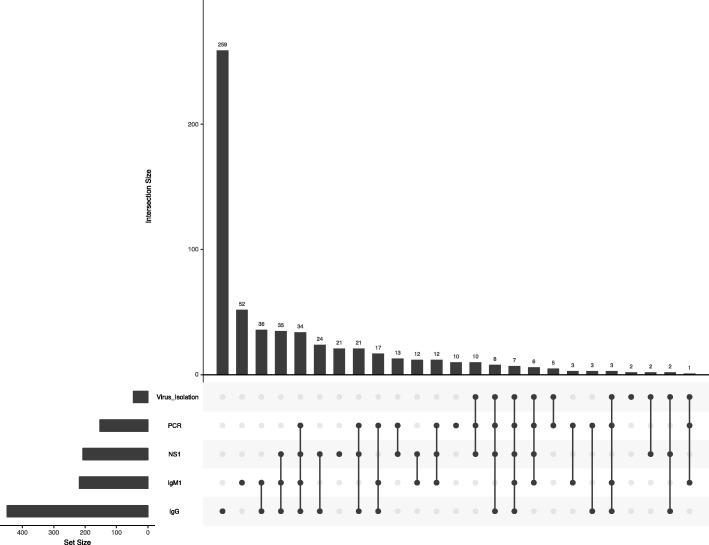
Matrix layout representing all combination of laboratorial diagnostic methods performed to study dengue fatal cases. Vertical bars indicate the number of individuals who were tested using each combination of techniques sorted by size. In the bottom of bars, dark circles in the matrix indicate when a combination of techniques was done. The bar chart on the left indicates the frequency of each technique employed

A total of 1047 dengue suspected fatal cases, representative from the North, Northeast, Midwest and Southeast regions of Brazil, were received and analyzed from 1986 to 2015, and 34.3% (359/1047) were confirmed as dengue by using any of the viral, molecular and serological diagnostic laboratory tests available in the routine of the Laboratory. Due to some samples volume restriction, not all samples were tested by all techniques, Table 1. All combinations of laboratorial diagnostic methods performed for the analysis of the dengue fatal cases are show on Fig. 1.

**Table 1:** Laboratorial diagnosis on dengue suspected fatal cases (*n =* 1047) confirmation in Brazil, 1986–2015

Diagnostic test	Sample		Total (%)
	Acute (< 7 days of illness) Positive/Tested (%)	Convalescent (≥7 days of illness) Positive/Tested (%)	
Virus isolation	46/768 (6.0)	Not done	46/768 (6.0)
RT-PCR	142/774 (18.3)	11/112 (9.82)	153/886 (17.3)
MAC-ELISA	120/489 (24.5)	42/113 (37.2)	162/602 (26.9)
IgG-ELISA	261/345 (75.6)	57/73 (78.1)	318/418 (76.0)
NS1-ELISA	120/415 (28.9)	24/93 (25.8)	144/508 (28.3)

The contribution of each method on the fatal cases confirmed were, as follows: DENV was isolated in 15.2% (46/302) of the confirmed cases after inoculation into C6/36 cells, and nested RT-PCR contributed in 46.5% (153/329) of the confirmed cases. The infecting serotype was more often identified by molecular detection and/or virus isolation in cases presenting 2 to 5 days of illness. The real time RT-PCR contributed confirming the infection on 60.5% (78/129) of the dengue fatal cases. The overall case confirmation by using the NS1 antigen ELISA was 67.2%, (207/308), in cases with up to 7 days of disease, but we observed positivity in convalescent samples, as well. The anti-DENV IgM antibody detection rate was 65.1% (218/335) on the dengue confirmed fatal cases Immunohistochemistry contribution was by confirming 59.9% (18/34) of the fatal cases by analyzing the paraffin embedded tissue samples available.

The highest percentage of confirmed cases were from the Southeast region, predominantly from the state of Rio de Janeiro, with 36.7% (132/359) of the cases, although fatal cases in the states of Espírito Santo, Goiás, Mato Grosso, Mato Grosso do Sul and Rio Grande do Norte were also reported. From the suspected fatal cases of dengue, 43.3% (447/1031) were female and 56.6% (584/1031) was male. The female to male confirmation ratio was 1:1.08 (171:186). We did not find a relationship between gender and the evolution to dengue fatal outcome (Table 2). In 16 cases, gender could not be defined due to the lack of information, dubious names or use of patients’ initials.

**Table 2:** Logistic models with logit links of epidemiological, virological and immunological variables influence on dengue mortality

Variable (Factor vs.)	Factor	*n* (fatal/non-fatal)	OR (95% CI)	*p* value
Gender	**Male**	**2165 (97/2068)**	1.12 (0.88–1.44)	0.42
Female vs				
*n* = 2279 (91/2188)				
Serotypes	**DENV-2**	**1047 (83/964)**	**5.67 (3.823–8.68)**	**< 0.0001**
DENV-1 vs.	**DENV-3**	**1279 (62/1217)**	**3.36 (2.23–5.50)**	**< 0.0001**
*n* = 1405 (21/1384)				
	**DENV-4**	**802 (23/779)**	**1.95 (1.17–3.23)**	**0.029**
DENV-2 vs.	**DENV-3**	**1279 (62/1217)**	**0.59 (0.42–0.83)**	**0.0024**
*n* = 1047 (83/964)				
	**DENV-4**	**802 (23/779)**	**0.34 (0.21–0.54)**	**< 0.0001**
DENV-3 vs.	**DENV-4**	**802 (23/779)**	**0.58 (0.35–0.93)**	**0.0281**
*n* = 1279 (62/1217)				
Age (years old)	**0–15**	**710 (38/672)**	**1.74 (1.17–2.60)**	**0.021**
16–30 vs.	**31–50**	**1205 (44/1161)**	1.16 (0.79–1.71)	0.522
*n* = 1075 (34/1041)				
	**51–96**	**604 (53/551)**	**2.94 (2.04–4.30)**	**< 0.0001**
0–15 vs.	**31–50**	**1205 (44/1161)**	0.66 (0.43–1.04)	0.07
*n* = 710 (38/672)				
	**51–96**	**604 (53/551)**	**1.68 (1.11–2.61)**	**0.017**
31–50 vs.	**51–96**	**604 (53/551)**	**2.53 (1.68–3.86)**	**< 0.0001**
*n* = 1205 (44/1161)				
Immune Responses	**Secondary**	**293 (74/219)**	0.97 (0.71–1.34)	0.89
Primary				
*n* = 265 (67/198)				

To calculate each logistic GLM, Death/Alive outcome was coded as binary variable. Odd ratios (COR), 95% confidence intervals (95%CI) and *P*-values were calculated using one GLM for each studied variable separately. Values highlighted in bold presented: COR > 1, values of OR contained into the 95%CI range and *p*-values < 0.05

**Fig. 2:**
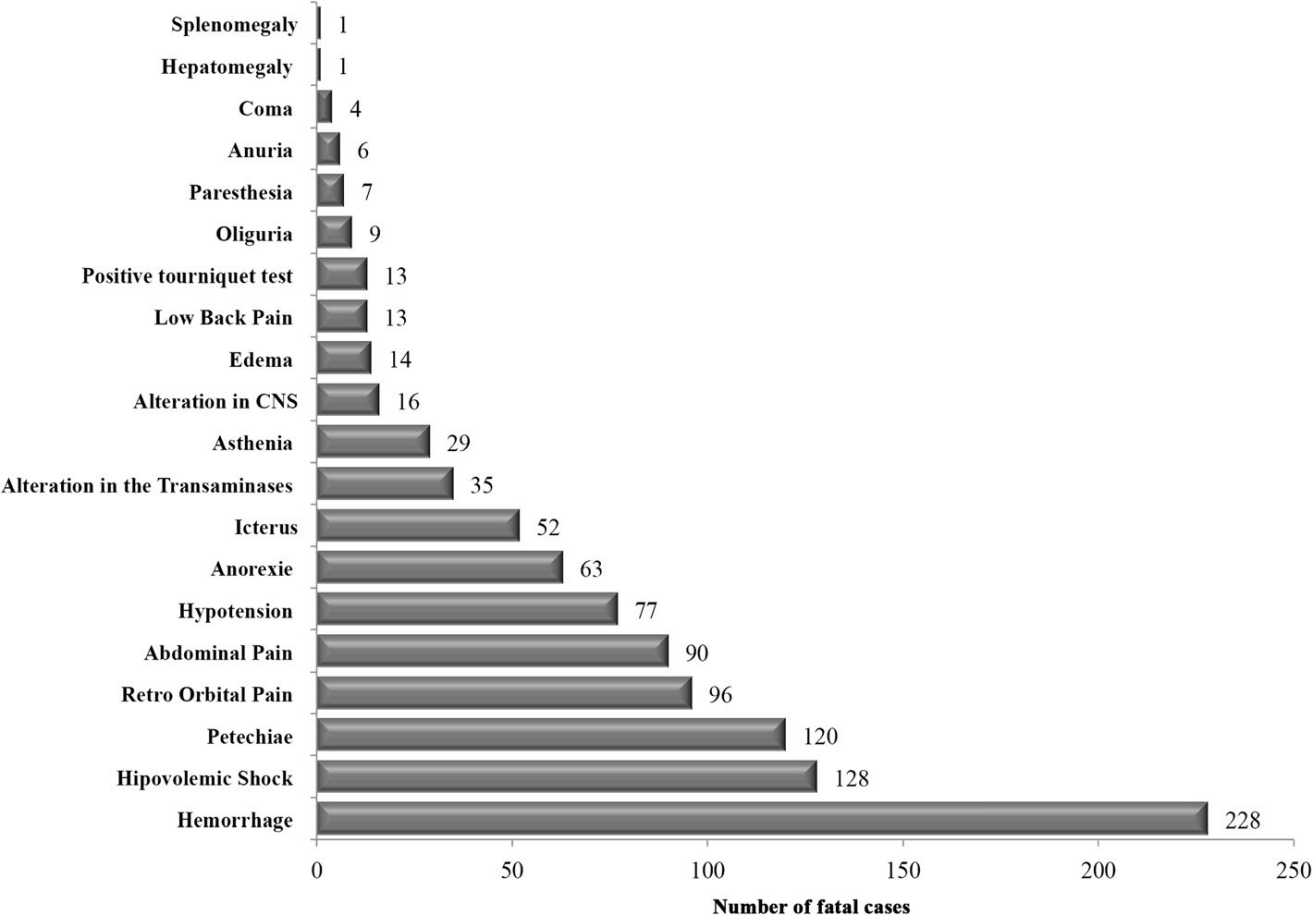
Signs and symptoms associated with severe dengue reported in the fatal cases (*n* = 359) analyzed in this study

Most patients developed systemic symptoms such as fever, myalgia, nausea, headache, and malaise. Hypovolemic shock was present in 137 (39.1%) of the patients and thrombocytopenia in 125 (35.7%). Hypotension was observed in 77 (22.0%) cases and abdominal pain in 93 (26.0%). Hepatomegaly was found in 19 (5.4%) patients and pleural effusion in 24 (6.8%). Coma and splenomegaly were rare (4 and case 1, respectively). The occurrence of hemorrhagic manifestations was also observed. Petechia were observed in 120 (34.3%) cases, epistaxis in 54 (15.4%), gingival bleeding in 46 (13.1%), non-specified bleeding in 43 (12.3%), haematemesis in 23 (6.5%), hematuria in 18 (5.1%) and a positive tourniquet test in 13 (3.7%). Irritability was reported in two cases, profuse perspiration and tachycardia in three cases; chills, cough, dizziness and neck stiffness in five cases. The clinical manifestations reported in 359 fatal cases with data available, during the period is shown in Fig. 2.

All four DENV serotypes were detected in the period: DENV-1 (11.1%, 21/189), DENV-2 (43.9%, 83/189), DENV-3 (32.8%, 62/189) and DENV-4 (13.7%, 26/189). Analyzing the influence of serotype in mortality, we observed that DENV-2 was more associated with fatal cases, followed by DENV-3 and DENV-4, Table 2.

Dengue infection was confirmed in 47.4% (72/152) cases were 0–15 years old, 44.0% (81/184) cases aged 16–30 years, 49.1% (105/214) cases with 31–50 years and 46.8% (110/235) cases with 51–96 years. Unfortunately, information on the age of 216 cases has not been described. We also observed that compared to 16–30, 0–15 and 31–50 years old group individuals ranging from 51 to 96 years old was more associated with fatal outcomes. Additionally, children age group (0–15 years old) was more associated to fatal outcomes compared to 16–30 years old group, Table 2.

Evaluating the association between dengue serotype and age, we observed that, consistently among the DENV 1 to 4 cases, the 51–96 years old group presented increased odds to present fatal cases compared to other age groups. Additionally, we observed increased odds in 0–15 years old groups compared to 16–30 groups for DENV-2 and DENV-3. Similarly, 31–50 years old groups had increased odds compared to 0–15 years old group for DENV-1 and DENV-3 (Table 3).

**Table 3:** Logistic models with logit links of association between serotype and age leading to the evolution to a dengue fatal outcome

Variable Factor (vs.)	Factor	*n* (fatal/non-fatal)	OR (95% CI)	*p value*
Serotype (Age group compared)	Age (years old)			
*DENV-1 (16–30 years old)*				
*n* = 309 (03/306)	**0–15**	**169 (05/164)**	3.13 (0.95–11.59)	0.122
	**31–50**	**314 (05/309)**	1.65 (0.50–6.10)	0.495
	**51–96**	**162 (7/155)**	**4.61 (1.55–16.33)**	**0.028**
*DENV-1 (0–15 years old)*				
*n* = 169 (05/164)	31–50	314 (05/309)	0.53 (0.14–1.92)	0.52
	51–96	162 (7/155)	1.47 (0.46–5.07)	0.32
*DENV-1 (31–50 years old)*				
*n* = 314 (05/309)	51–96	162 (7/155)	2.79 (0.88–9.56)	0.08
*DENV-2 (16–30 years old)*				
*n* = 309 (11/306)	**0–15**	**130 (26/104)**	**3.61 (1.97–6.91)**	**0.0006**
	**31–50**	**263 (16/247)**	1.07 (0.56–2.12)	0.864
	**51–96**	**121 (19/102)**	**3.08 (1.62–6.06)**	**0.005**
*DENV-2 (0–15 years old)*				
*n* = 130 (26/104)	**31–50**	**263 (16/247)**	**0.3 (0.15–0.57)**	**0.0003**
	**51–96**	**121 (19/102)**	0.85 (0.44–1.62)	0.62
*DENV-2 (31–50 years old)*				
*n* = 263 (16/247)	**51–96**	**121 (19/102)**	**2.88 (1.76–6.65)**	**0.003**
*DENV-3 (16–30 years old)*				
*n* = 348 (17/331)	**0–15**	**247 (04/243)**	0.31 (0.11–0.73)	0.037
	**31–50**	**369 (18/351)**	0.99 (0.56–1.77)	0.997
	**51–96**	**174 (17/157)**	**2.11 (1.17–3.80)**	**0.036**
*DENV-3 (0–15 years old)*				
*n* = 247 (04/243)	**31–50**	**369 (18/351)**	**3.22 (1.18–11.24)**	**0.036**
	**51–96**	**174 (17/157)**	**6.79 (2.46–23.94)**	**0.0007**
*DENV-3 (31–50 years old)*				
*n* = 369 (18/351)	**51–96**	**174 (17/157)**	**2.11 (1.05–4.22)**	**0.036**
*DENV-4 (16–30 years old)*				
*n* = 225 (03/222)	**0–15**	**136 (03/133)**	1.66 (0.41–6.75)	0.54
	**31–50**	**259 (05/254)**	1.46 (0.44–5.40)	0.609
	**51–96**	**147 (10/137)**	**5.4 (1.95–18.51)**	**0.011**
*DENV-4 (0–15 years old)*				
*n* = 136 (03/133)	**31–50**	**259 (05/254)**	0.88 (0.21–4.34)	0.86
	**51–96**	**147 (10/137)**	3.26 (0.97–14.78)	0.08
*DENV-4 (31–50 years old)*				
*n* = 259 (05/254)	**51–96**	**147 (10/137)**	**3.71 (1.29–12.11)**	**0.018**

To calculate each logistic GLM, Death/Alive outcome was coded as binary variable. Odd ratios (COR), 95% confidence intervals (95%CI) and *p*-values were calculated using one GLM for each studied variable separately. Values highlighted in bold presented: OR > 1, values of OR contained into the 95%CI range and *p*-values < 0.05

**Fig. 3:**
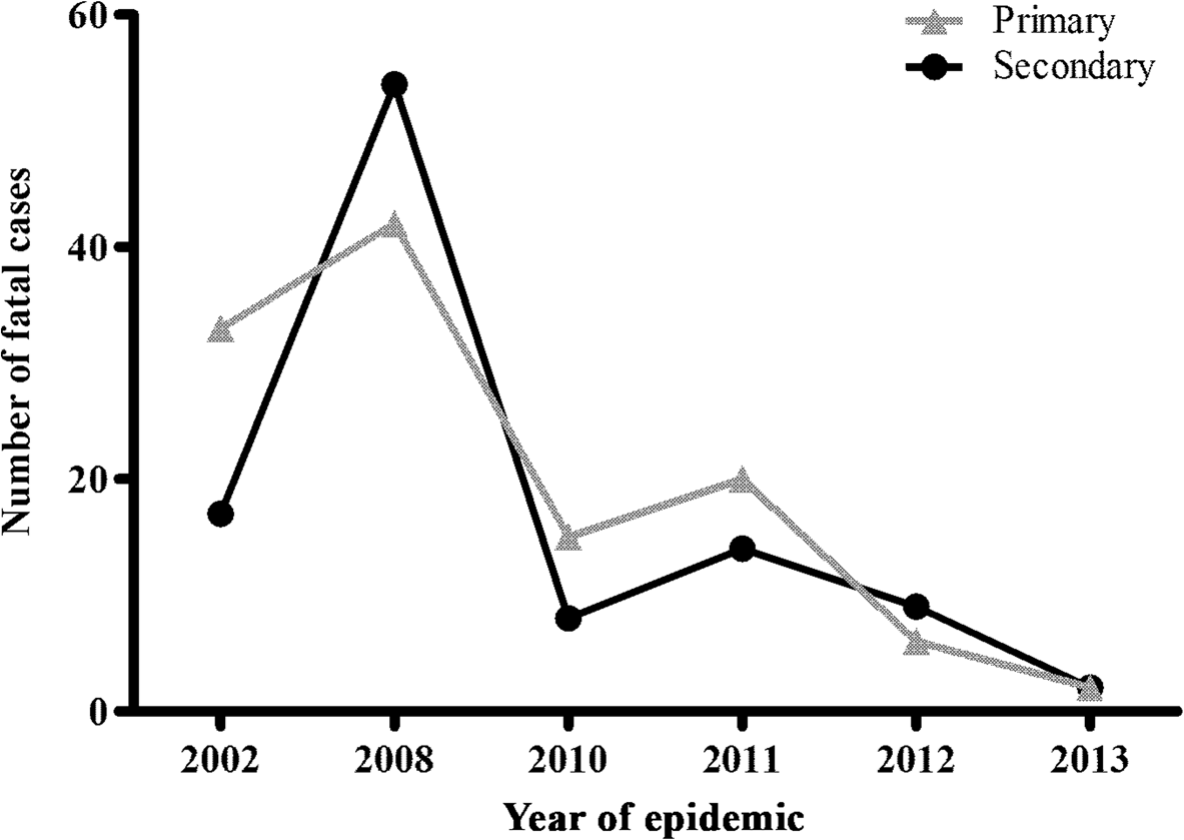
Dengue fatal cases (*n* = 222) immune response occurred during epidemics in Brazil

The patients’ immune response was characterized by IgG-ELISA in 300 fatal cases and, primary infections (59.3%; 178/300) were more often observed than secondary ones (40.6%; 122/300; *p* = 0.001). However, we did not find a relation between the immune response and the evolution to death (Table 2). Except by the year 2008, when most deaths were due to secondary infections, during the most expressive epidemic years 2002, 2010, 2011, 2012 and 2013, fatal cases were mainly due to primary ones (*p* = 0.038), Fig. 3. 46.2% (31/67) of DENV-3 cases were characterized as primary infection while 62.1% (46/74) of DENV-2, as secondary ones. No association was observed among the type of infection (primary or secondary), serotype and the evolution to a fatal outcome, Table 4. Nevertheless, considering the age factor, children 15 years old and under, presenting a secondary infection had almost a 4-fold risk of death, Table 4.

**Table 4:** Logistic models with logit links of association of dengue serotype and Age with immune response that lead to the evolution to a dengue fatal outcome

Variable Factor (vs.)	Factor	*n* (fatal/non-fatal)	OR (95% CI)	*p* value
Serotype	**Immune Responses**			
‘n’ Primary Responses				
DENV-1	**Secondary**	**67 (5/62)**	0.38 (0.14–0.96)	0.095
*n* = 57 (10/47)				
DENV-2	**Secondary**	**140 (46/94)**	0.83 (0.49–1.41)	0.558
*n* = 62 (23/39)				
DENV-3	**Secondary**	**82 (19/63)**	1.04 (0.60–1.79)	0.904
*n* = 138 (31/107)				
DENV-4	**Secondary**	**4 (04/00)**	–	–
*n* = 8 (03/05)				
Age	**Immune Responses**			
(years old) ‘n’ Primary Responses				
0–15	**Secondary**	**46 (24/22)**	**3.93 (1.86–8.62)**	**0.003**
*n* = 46 (10/36)				
16–30	**Secondary**	**63 (10/53)**	0.6 (0.28–1.26)	0.267
*n* = 63 (15/48)				
31–50	**Secondary**	**100 (19/81)**	0.95 (0.52–1.76)	0.89
*n* = 86 (17/69)				
51–96	**Secondary**	**51 (18/33)**	1.83 (0.89–3.83)	0.172
*n* = 37 (18/19)				

To calculate each logistic GLM, Death/Alive outcome was coded as binary variable. Odd ratios (COR), 95% confidence intervals (95%CI) and *p*-values were calculated using one GLM for each studied variable separately. Values highlighted in bold presented: OR > 1, values of OR contained into the 95%CI range and *p*-values < 0.05

In 2002, an epidemic caused by DENV-3 was reported and 97.7% (43/44) of the fatal cases investigated in that year were due to that infecting serotype. On the other hand, the 2008 epidemic was mainly caused by DENV-2, the infecting serotype identified in 89.0% (60/67) of the fatal cases investigated in this study. In 2010, DENV-1 (41.7%, 5/12) and DENV-2 (58.3%, 7/12) were the infecting serotypes identified, while the latter was responsible for 82.3% (14/17) of cases studied in 2011. In 2012, two fatal cases were confirmed, one by DENV-2 and one by DENV-3. In 2013, all nine fatal cases confirmed were due to DENV- 4. During the years of 2014 and 2015, three fatal cases were confirmed by DENV-4 and only one case by DENV-1, occurred in 2014, Fig. 4.

**Fig. 4:**
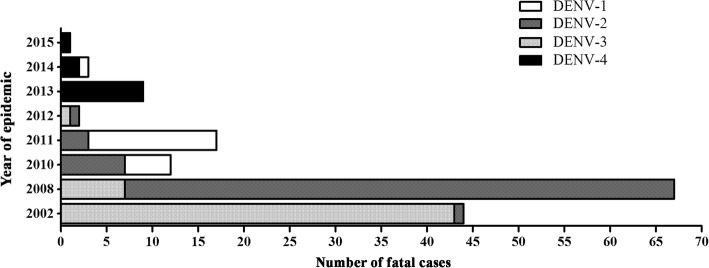
Dengue infecting serotypes identified in the fatal cases (*n* = 155) occurred during epidemics in Brazil

**Fig. 5:**
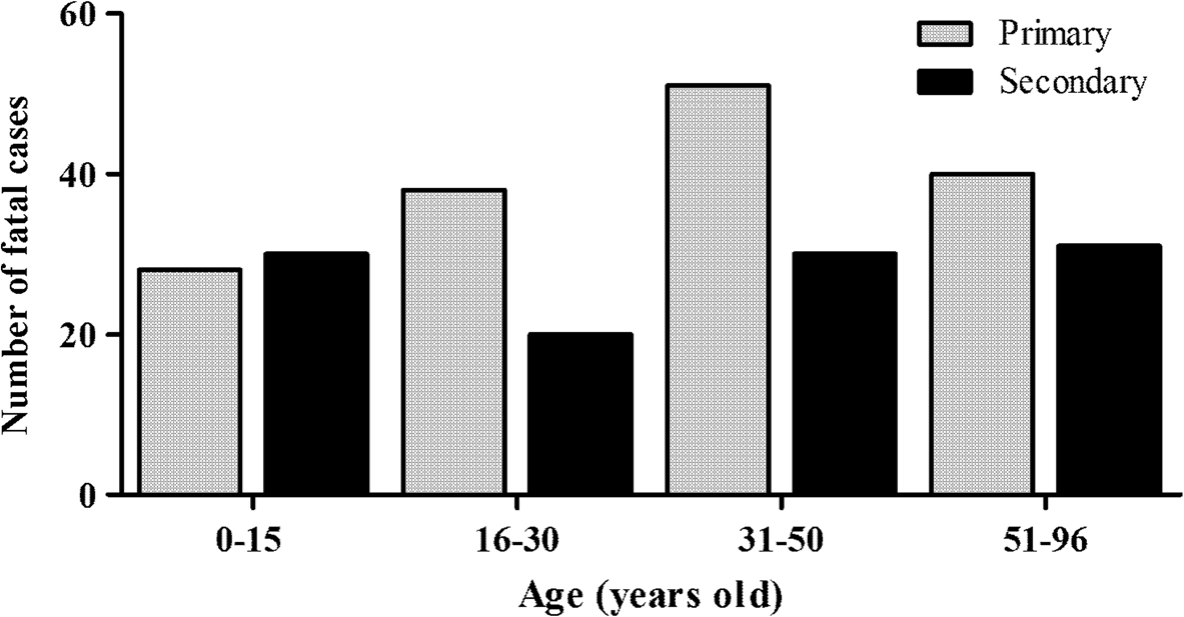
The relationship between age and immune response of dengue fatal cases (*n* = 205) analyzed in Brazil, 1986–2015

**Fig. 6:**
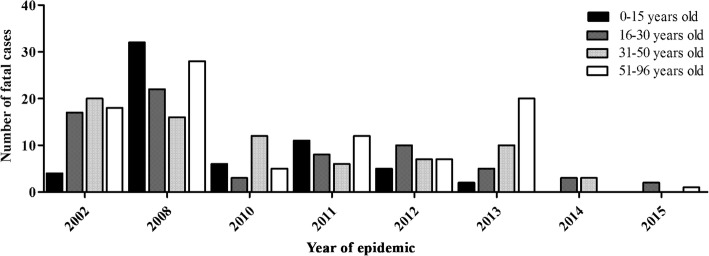
Age distribution of dengue fatal cases (*n* = 164) occurred during epidemics in Brazil

The fatal cases’ age ranged from 0 to 96 years old, with a predominance of positive cases in children 0–15 years old and between 51 and 96 years old (25.6%, 42/164). Fatal outcome on age groups between 16 to 30 and 31–50 years occurred in 24.4% (40/164) of the cases each. In 195 cases the age was unknown. Despite the prevalence of secondary infections on children 0–15 years old, no significant differences were observed (*p* = 0.350), Fig. 5. In the epidemic years of 2002, 2010, 2012, 2013, 2014 and 2015, the majority of the fatal cases occurred adults, however in 2008 and 2011, increased fatal cases on children 15 years old and under, were observed (*p* = 0.045), Fig. 6. No significant differences were observed when age groups and the patient’s immune response were compared, considering the epidemics of 2002, 2008, 2010, 2012, 2013, 2014 and 2015.

## Discussion

Over the past 30 years, more than 5202 deaths from dengue were reported in Brazil, and the disease has become a serious public health problem in several states [31]. During this period, the Laboratory of Flaviviruses (LABFLA) IOC/ FIOCRUZ, a Regional Reference Laboratory for Dengue and Yellow Fever diagnosis based on Rio de Janeiro, Southeast region of Brazil, received suspected dengue cases, meeting the requirements of the Ministry of Health to monitor the disease in the country.

In 1986, with the introduction of DENV-1 in Rio de Janeiro [32], the disease was established causing an explosive epidemic in a naïve population and spread to other Brazilian states. In 1990, DENV-2 was also isolated in Rio de Janeiro and this scenario resulted in the occurrence of the first cases of severe disease [33]. In December 2000, another serotype, DENV-3, was detected in Rio de Janeiro and was responsible until then for the largest and most severe dengue epidemic ever described in the country and in the American continent, not only due to the high number of reports and fatal cases [34–36]. The reemergence of DENV-2 in 2007 characterized a dramatic increase in the number of severe cases and deaths in children 15 years old and under [37]. In 2009, a new high-transmission cycle of DENV-1 began in Brazil, with more than one million probable cases and 656 deaths reported in 2010 and the occurrence of deaths in patients with comorbidities [38]. In July 2010, DENV-4 was isolated in Roraima [39] and in 2011 this serotype spread to other states.

Since 2014, Brazil has experienced triple epidemics caused simultaneously by DENV, chikungunya virus (CHIKV) and zika virus (ZIKV), hampering the clinical differential diagnosis, as those arboviruses share common signs and symptoms. Despite the zika epidemic occurred, in 2015, a total of 1,649,008 probable dengue cases and 863 deaths, mainly caused by DENV-1 and DENV-4, were reported in Brazil [40].

About 75% of dengue infections are known to be clinically unapparent or mildly symptomatic [2]. More severe cases may be characterized by hemorrhagic events, thrombocytopenia and increased leakage of fluid and shock that can evolve and lead to death within 12–36 h [41].

The 1997 World Health Organization (WHO) criteria used to classify dengue infections as dengue fever, dengue hemorrhagic fever, and dengue shock syndrome [42]. However, due its not always reliable usage to classify patients with a more severe disease, a revised classification (dengue without warning signs, dengue with warning signs, and severe dengue) was suggested and evaluated [43–45], aiming to timely manage the patient and avoid increased severity and fatal outcome.

Among the hypothesis proposed to explain the wide spectrum of dengue clinical manifestations are the samples virulence, sequential infections and multiple causality characteristics represented by individual (age sex, race, nutritional status, co-morbidities), epidemiological (immunity, competence and vector density, hyperendemicity, interval between infections by different serotypes) and viral factors, such as the virulence of the circulating strain and/or strain genotype [46].

In this study, no differences were observed when we analyzed the fatal outcome in relation to the patients gender, corroborating the findings by Wang [47] and Thomas [48]. On the other hand, a study carried out by Anders [49] in Vietnam, associated a greater risk for disease severity in females, with 1.57 higher risk to evolve to death than the males. Similarly, Sam [7] reported fatal cases in 9 out of 10 females analyzed. The study by Araujo [50] in 84 fatal cases reported death in 54% of male. Moreover, Leo [51] reported that 67.9% of men infected with dengue evolved to death in comparison to women.

Fever, myalgia, nausea, headache, malaise, hypovolemic shock, thrombocytopenia, abdominal pain and hypotension were described on the fatal cases analyzed. Less commonly observed were hepatomegaly and pleural effusion. Only four cases went into coma and only one had splenomegaly. Hemorrhagic manifestations more frequently observed were petechiae, epistaxis, gingival bleeding, hematemesis and hematuria (Fig. 2), corroborating observations in Cuba [52, 53], Singapore [54], Malaysia [7], India [55] and Taiwan [56].

Whitehorn & Simmons [57] reported that age is an important factor for severe dengue and death, and vietnamese children up to 5 years old were four times more likely to have a more severe disease than the 11–15 year-old group. On the other hand, García-Rivera & Rigau-Pérez [58] demonstrated that the elderly had 6 times more risk of death than young adults, and almost 2 times more than infants. In our analysis, during the entire study period, we observed the age groups 0–15 and 51–96 years old presented 1744 (95%CI: 1.173–2.600) and 2.945-fold (95%CI: 2.038–4.294) increased risk of death, respectively (Table 2).

When we analyzed epidemic and inter-epidemic periods, it was shown that up to 2006, the highest rates of dengue and severe dengue in Brazil occurred in patients over 15 years old. This same pattern was observed in an epidemic in 2010 in Puerto Rico where adults accounted for 49.7% of severe cases and fatal cases of dengue (92.5%) [59]. In Pakistan, a higher number of severe cases were observed in individuals over 30 years old in 2011 [60].

The initial pattern of severe cases in young adults presented significant changes in recent years in Brazil. In 2007, increased hospitalization rates and severe dengue in children 15 years old and under were reported, similar to the observations on Southeast Asia [61, 62]. These data corroborate those found in our study, since we observed that during the 2008 epidemic caused by DENV-2, fatal cases in children under 15 years old were more frequent and not observed in other epidemics and children aged 0–15 years infected with DENV-2 had increased odds of cases evolve to death (Table 3, Fig. 3-6). As the co-circulation of several DENV serotypes increases in Brazil, adults are less likely to remain susceptible to infection [63].

Several studies have shown that secondary infections were related to increased risk of severe dengue and death [14, 52, 64–66]. A study by Nisalak [67] found that secondary infections had a five-fold increased risk for the occurrence of dengue haemorragic fever than primary infections. In a cohort of 97 pediatric patients in India, the evolution of the disease severity was greater in secondary infections and in approximately one third of primary infections [68]. In our study, fatal cases due to primary infections were more significantly observed than secondary ones (*p* = 0.001). The analysis of fatal cases occurred in 2002 by DENV-3 also reported a higher frequency of primary infections [69]. The highest number of fatal cases due to secondary cases was a characteristic of the DENV-2 epidemic. Furthermore, children 0–15 years old presenting secondary infection showed a 4-fold increased risk (95%CI: 1.863–8.620) to a fatal outcome (Table 4). However, a study in children in Thailand did not point out a relationship between the disease severity and immune response [47]. A recent systematic review on dengue mortality, reported fatal cases to be more common in individuals presenting secondary infections and none of the reports associated deaths to primary infection [70].

It has been postulated that the disease severity may be due to the genetic and antigenic variations of the different DENV strains, as the genetic evolution of the virus within each serotype may give rise to more virulent strains [71, 72]. Despite that, any of the four DENV serotypes may lead to severe and fatal cases and a hyperendemic scenario, with the co-circulation of distinct DENV serotypes may increase the chances of more severe disease [4].

DENV-2 and DENV-3 are the serotypes most commonly associated with fatal cases. In our study, DENV-2 was identified in 43.9% (83/189) of the fatal cases and was associated with a 5-fold increased risk (95% CI: 3.829–8.678) of death when compared to DENV-1. Similarly, DENV-3 caused 32.8% (62/189) of deaths and presented an increase of 3-fold (95% CI: 2.233–5.495) for death (Table 2). Furthermore, previous studies have reported that DENV-2 secondary infections, mainly by the Asian genotype which circulates in Brazil, has led to an increase in severe cases such as hemorrhagic fever and dengue shock syndrome [52, 65, 73]. In fact, the mortality rate was twice higher after the introduction of the new lineage of the Asian DENV-2 (Lineage II) in 2007 in Brazil [61, 74–76]. DENV-3 circulating in Brazil belongs to genotype III, also of Asian origin and it has been associated to the severe disease occurred in 2002 [77].

In Thailand, a study reported a higher frequency of DENV-2 (35%) and DENV-3 (31%) cases in children during 20 years of investigation [67] and those serotypes were associated to severe cases in children up to 15 years old, in comparison to DENV-1 cases [78]. In our study, children up to 15 years old infected with DENV-2 had nearly 4-fold increased risk of dying when compared to the same age group in the other serotypes, (Table 3). However, a relationship was also observed on elderly who were infected by any of the serotypes, Table 3. In a DENV-3 epidemic occurred in Havana, Cuba during 2001–2002, 12,889 cases and 81 DHF cases were reported [53]. However, a study in Puerto Rico in 2010 reported that DHF patients were more likely to have been infected by DENV-4 than DENV-1 [59]. In this study, DENV-2 infected individuals had almost 2 times (95%CI: 1.177–3.228) more risk of dying when compared to those infected by DENV-1 (Table 2).

DENV-1 followed by DENV-2 infections were associated with outbreaks of hemorrhagic fever [79]. However, other sequential infections, such as DENV-3 followed by DENV-2, DENV-1 by DENV-3 and DENV-2 by DENV-3 in El Salvador (2000), Cuba (2000–2001) and Brazil (2001–2002), respectively, were associated with severe disease [80]. In DENV-2 infected children previously infected by DENV-3, the occurrence of a more severe disease was also reported [81].

## Conclusions

This study demonstrates that the cause of dengue mortality in Brazil is multifactorial and, although there is much information on the disease epidemiology, information on the causes of dengue mortality is still scarce. Despite all epidemics occurred in the past three decades, the increased severity still leads to a significant number of fatal cases. The analysis performed here, demonstrates how host and viral factors play a role in the disease outcome. Moreover, with the current troublesome clinical differential diagnosis, the performance of distinct laboratorial diagnostic methods is imperative for the disease surveillance in the context of endemic arboviruses scenario.

The study has limitations and those include, in some cases, the quality of the record resulting in the lack of some clinical and/or demographical information, as well the full course of disease during hospitalization. For instance, the analysis of signs and symptoms, infecting serotype, immune response and demographic characteristics were performed only on those cases with data and information available. Dengue cases are still underreported in Brazil and the need of improvement in the proper filling of report forms has been stressed [81]. Despite this, each case record and laboratorial diagnosis results were extensively reviewed and discussed by a physician and laboratory personnel. Another limitation includes the insufficient volume of the samples in some cases for further characterization. Other pathogens were not investigated for, and thus deaths due to bacterial pathogens could not be excluded. Despite the convenience sampling used in this study, the strength of this study lies on the analysis of a considerable number of cases investigated, one of the largest reported so far in the literature and from a comprehensible period (30 years), involving dengue epidemics occurred by the four distinct serotypes in Brazil. Moreover, as a Reference Laboratory, cases were primarily sent for investigation in a time fashion manner.

## Abbreviations

CHIKV: Chikungunya virus
CI: Confidence interval
DENV: Dengue virus
ELISA: Enzyme –linked immunosorbent assay
GLM: Logistic generalized linear models
HI: Haemaglutination inhibition test
ICT: Immunochromatographic test
IFAT: Indirect fluorescent antibody test
IgG: Immunoglobulin G
IgG-ELISA: Immunoglobulin G antibody detection ELISA
IgM: Immunoglobulin M
LABFLA: Laboratory of Flavivirus
MAC-ELISA: Immunoglobulin M antibody capture ELISA
OR: Odd ratio
qRT-PCR: Real-time Reverse Transcriptase PCR (TaqMan) assay
RT-Nested-PCR: Reverse transcriptase-nested polymerase chain reaction
RT-PCR: Reverse transcriptase polymerase chain reaction
WHO: World Health Organization
ZIKV: Zika virus
